# Systemic Amyloidosis in England: an epidemiological study

**DOI:** 10.1111/bjh.12286

**Published:** 2013-03-11

**Authors:** Jennifer H Pinney, Colette J Smith, Jessi B Taube, Helen J Lachmann, Christopher P Venner, Simon D J Gibbs, Jason Dungu, Sanjay M Banypersad, Ashutosh D Wechalekar, Carol J Whelan, Philip N Hawkins, Julian D Gillmore

**Affiliations:** 1 Department of Medicine, UK National Amyloidosis Centre, Royal Free and University College Medical SchoolLondon, UK; 2 Department of Medicine, UCL Centre for Nephrology, Royal Free and University College Medical SchoolLondon, UK; 3 UCL Division of Medicine, Research Department of Infection and Population HealthLondon, UK

**Keywords:** amyloidosis, incidence, death certificate, survival

## Abstract

Epidemiological studies of systemic amyloidosis are scarce and the burden of disease in England has not previously been estimated. In 1999, the National Health Service commissioned the National Amyloidosis Centre (NAC) to provide a national clinical service for all patients with amyloidosis. Data for all individuals referred to the NAC is held on a comprehensive central database, and these were compared with English death certificate data for amyloidosis from 2000 to 2008, obtained from the Office of National Statistics. Amyloidosis was stated on death certificates of 2543 individuals, representing 0·58/1000 recorded deaths. During the same period, 1143 amyloidosis patients followed at the NAC died, 903 (79%) of whom had amyloidosis recorded on their death certificates. The estimated minimum incidence of systemic amyloidosis in the English population in 2008, based on new referrals to the NAC, was 0·4/100 000 population. The incidence peaked at age 60–79 years. Systemic AL amyloidosis was the most common type with an estimated minimum incidence of 0·3/100 000 population. Although there are various limitations to this study, the available data suggest the incidence of systemic amyloidosis in England exceeds 0·8/100 000 of the population.

Systemic amyloidosis is a disorder of protein folding in which certain proteins auto-aggregate to form insoluble amyloid fibrils that accumulate in the extracellular space and impair organ function. Amyloidosis is a progressive and usually fatal disease that has been loosely estimated to cause about 0·5–1·0 deaths per thousand in the UK (Pepys, 2001), mostly of systemic AL amyloidosis type. However, there are almost no published epidemiological studies of amyloidosis in the medical literature; the most comprehensive having been a study conducted at the Mayo Clinic of the general population residing in the surrounding area of Olmstead County, USA (Kyle *et al*, 1992). They reported an incidence of AL amyloidosis of 5·1–12·8 per million person-years, using data from a centralized system recording virtually all medical, surgical and pathological diagnoses of the county residents. Twenty one individuals from Olmstead County were diagnosed with AL amyloidosis between 1952 and1992. The overall age- and sex-adjusted annual incidence rate was reported to be 8·9 per million person-years. The authors extrapolated that approximately 2225 new cases may occur annually across the USA, but this has not been verified.

The UK National Amyloidosis Centre (NAC) provides a diagnostic and treatment advisory service that is available free of charge at the point of delivery to National Health Service (NHS)-entitled patients with proven or suspected amyloidosis, and is the only specialist centre in the country. Data on individuals referred to our centre are held on a comprehensive database that effectively serves as a national registry of the disease. This database has enabled the natural history and response to therapeutic interventions of the various amyloidosis syndromes to be studied in large cohorts of patients.

Studies using data from death certificates have been conducted in several rare diseases to estimate the burden of mortality (Cotch *et al*, 1996; Thomas *et al*, 2010; Marin *et al*, 2011) and indirectly estimate the incidence of disease. Systemic amyloidosis lends itself to this approach because it is an incurable and usually rapidly fatal disorder (Gillmore *et al*, 2006, 2009; Lachmann *et al*, 2007; Kumar *et al*, 2010); it has specific pathological features enabling definitive diagnosis through biopsy, and which are overt in hitherto undiagnosed cases at autopsy. Further, given the requirement for histological diagnosis, it is probable that false positive recording of amyloidosis on death certificates is exceptionally rare.

We estimate here the incidence of amyloidosis in England based on an analysis of two data sets: reported death certification from the Office of National Statistics (ONS) and information on referrals and deaths held on the NAC database.

## Methods

### Office of National Statistics (ONS) death certificate data

The number of deaths registered in each calendar year for people living in England and the proportion of those deaths in whom the word ‘amyloidosis’ appeared anywhere on the death certificate, as defined by the International Classification of Diseases ninth revision (ICD-9) code for the year 2000 and tenth revision (ICD-10) code from 2001 onwards (Table I), were obtained from the ONS. All data was fully anonymized.

**Table I:** Amyloidosis deaths – International Classification of Diseases Ninth (ICD-9) and Tenth Revision (ICD-10)

Cause of death	ICD-9 code
Amyloidosis	277·3
Cause of death	ICD-10 code
Non-neuropathic heredofamilial amyloidosis	E85·0
Neuropathic heredofamilial amyloidosis	E85·1
Heredofamilial amyloidosis, unspecified	E85·2
Secondary systemic amyloidosis	E85·3
Organ-limited amyloidosis	E85·4
Other amyloidosis	E85·8
Amyloidosis, unspecified	E85·9

### Data from the National Amyloidosis Centre database

All English residents diagnosed to have systemic amyloidosis between 2000 and 2008 whose details were held on the NAC databases were identified and their details were provided to ONS. Death data was returned from ONS for all matched individuals, including date and cause of death as listed in parts IA, IB, IC, and II of the respective death certificates. Matching of individuals between the ONS and NAC databases required agreement of name, date of birth and unique NHS number. The type of amyloid and date of diagnosis was obtained for each case from the NAC database and survival from the first assessment at NAC was calculated. Survival data were censored on 1 January 2012.

### Office of National Statistics estimate of population

Estimates of the population in England were taken from the ONS website ‘Mid-1971 to Mid-2010 Population Estimates: Quinary age groups for Constituent Countries in the United Kingdom; estimated resident population’ released on 21 December 2011 (available at: http://www.ons.gov.uk/ons/rel/pop-estimate/population-estimates-for-uk-england-and-wales-scotland-and-northern-ireland/population-estimates-timeseries-1971-to-current-year/index.html). Estimates are of the usually resident population on 30 June of the reference year and reflect administrative boundaries that were in place on that day.

### Ethics and statistics

The study was approved by the Royal Free Hospital ethics committee. Graph Pad Prism version 5 (Graphpad software Inc., San Diego, CA, USA) was used for statistical analyses. Patient survival was estimated by Kaplan–Meier Analysis using SPSS version 20 (IBM, New York, USA). The log-rank test was used to compare differences between stratified Kaplan–Meier survival curves. Statistical significance was achieved if *P* <0·05.

## Results

### Accuracy of death certificate data

Summary statistics on the total number of deaths attributable to systemic amyloidosis between 2000 and 2008 are shown in Table II. Amyloidosis appeared on the death certificates of 2543 English individuals during this period. There were 1143 deaths over the same period among patients with systemic amyloidosis who were registered on the NAC database, 903 (79%) of whom had amyloidosis reported on their death certificate. The proportion of patients registered on the NAC database for whom amyloidosis was stated on the death certificate did not vary significantly over the study period. However, there was an association between amyloid type and the frequency with which amyloidosis appeared on the death certificate (Table III, *P* ≤ 0·001); 83% of patients with AL amyloidosis had amyloidosis on their death certificate, compared to 74% of those with hereditary amyloidosis and *c*. 62% of those with AA and transthyretin amyloidosis.

**Table II:** Total number of deaths in England with amyloidosis recorded anywhere on the death certificate and total number of patients from England reviewed at the National Amyloidosis Centre (NAC) between 2000 and 2008

Year	2000	2001	2002	2003	2004	2005	2006	2007	2008	Total
Data from death certificates (ONS)										
Amyloidosis reported as the underlying cause of death (% of total certificates with amyloidosis stated)	128 (58)	154 (63·4)	171 (60·8)	153 (58·6)	165 (62·9)	197 (62·5)	174 (61·7)	208 (63·8)	217 (61·5)	1567 (61·6)
Amyloidosis anywhere on the death certificate	220	243	281	261	262	315	282	326	353	2543
Total deaths in England	503 024	497 878	500 795	504 127	480 716	479 678	470 326	470 721	475 763	438 3028
Proportion of deaths with amyloid mentioned on death certificate (per thousand deaths)	0·44	0·49	0·56	0·5	0·54	0·66	0·60	0·69	0·74	0·58
Data from NAC database										
Deaths in patients seen at NAC, *N*	66	84	98	110	116	144	151	157	217	1143
NAC deaths with amyloid on certificate, *N* (%)	55 (83·3)	69 (82·1)	82 (83·7)	87 (79·1)	87 (75)	119 (82·6)	118 (78·1)	116 (73·9)	170 (78·3)	903 (79·0)
Total deaths with amyloid on death certificate and confirmed NAC deaths with no mention of amyloid, *N*	231	258	297	284	291	340	315	367	400	2783
National proportion of deaths with amyloid from death certificate and NAC confirmed cases (per thousand deaths)	0·46	0·52	0·59	0·56	0·61	0·71	0·67	0·78	0·84	0·63
Patients with amyloid on death certificate not seen at NAC, *N* (%)	165 (75)	174 (71·6)	199 (70·8)	174 (66·6)	175 (66·8)	196 (62·2)	164 (58·2)	210 (64·4)	183 (51·8)	1640 (64·5)

ONS, Office of National Statistics; NAC, National Amyloidosis Centre.

**Table III:** Number of patients seen at the National Amyloidosis Centre with amyloid on their death certificate, stratified by amyloid fibril type

	AL amyloidosis		AA amyloidosis		Hereditary amyloidosis		Senile systemic amyloidosis	
Amyloid mentioned on death certificate	No	Yes	No	Yes	No	Yes	No	Yes
2000	4 (8)	46 (92)	6 (60)	4 (40)	1 (16·7)	5 (83·3)	0	0
2001	7 (10·3)	61 (89·7)	5 (38·5)	8 (61·5)	3 (100)	0	0	0
2002	12 (15·3)	72 (84·7)	3 (42·9)	4 (57·1)	1 (14·3)	6 (85·7)	0	0
2003	16 (18·4)	71 (81·6)	5 (29·4)	12 (70·6)	0	4 (100)	2 (100)	0
2004	16 (18·2)	72 (81·8)	6 (54·6)	5 (45·4)	2 (22·3)	7 (77·7)	5 (62·5)	3 (38·5)
2005	22 (18·1)	100 (81·9)	2 (14·3)	12 (85·7)	1 (12·5)	7 (87·5)	0	0
2006	23 (19·3)	96 (80·7)	6 (31·6)	13 (68·4)	4 (40)	6 (60)	0	3 (100)
2007	27 (22·3)	94 (77·7)	11 (47·8)	12 (52·2)	1 (16·7)	5 (83·3)	2 (28·6)	5 (71·4)
2008	33 (9)	139 (81)	6 (37·5)	10 (62·5)	6 (30)	14 (70)	2 (22·3)	7 (77·7)
Total (%)	160 (17·5)	751(82·4)	50 (38·5)	80 (61·5)	19 (26)	54 (74)	11 (37·9)	18 (62·1)

### Cause of death among patients with systemic amyloidosis

**Fig. 1:**
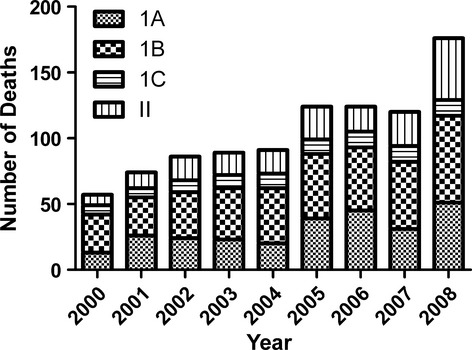
Position of amyloidosis on death certificates among patients who attended the National Amyloidosis Centre.

Among 903 patients who had amyloidosis on their death certificate and were followed for systemic amyloidosis at the NAC, amyloidosis was recorded in part 1A, 1B, and 1C of the death certificate in 261 (29%), 372 (41%) and 96 (10%) cases respectively. Amyloidosis was recorded in part 2, which identifies conditions contributing to the cause of death, in 174 (19%) individuals (Fig 1). The primary cause of death was probably directly related to amyloidosis among 127/174 (73%) such patients, and was listed as follows; 12 end-stage renal failure (ESRF), 22 heart failure, 56 sepsis, 14 multi-organ failure, 17 myeloma/lymphoma, five pulmonary embolism and one nephrotic syndrome. A further 32 (18%) deaths among these 174 individuals were possibly related to the amyloidosis and the primary causes were listed as follows; 17 cerebrovascular accidents (CVA), nine myocardial infarctions (MI), four sudden cardiac deaths, one ruptured abdominal aortic aneurysm and one bowel perforation. Only 15/174 (9%) deaths appeared unrelated to systemic amyloidosis, 14 from unrelated malignancies and one from chronic obstructive pulmonary disease (COPD).

Of the 240 individuals with systemic amyloidosis who were followed at the NAC but did not have amyloidosis stated on their death certificate, 179 (75%) had primary causes of death which were probably related to their amyloid. Among the remaining 61/240 (25%) patients the primary cause of death was listed as follows; 26 ischaemic heart disease or heart failure, 13 ‘other’ malignancy, 10 CVA, 3 COPD, two sepsis, and one each from drug overdose, end-stage renal failure, bowel obstruction, bowel ischaemia, pelvic abscess, peritonitis, and vasculitis. Taken together, these results suggest that amyloidosis contributed directly to death in more than 90% of patients with systemic amyloidosis.

### Estimate of deaths attributable to amyloidosis in England

**Fig. 2:**
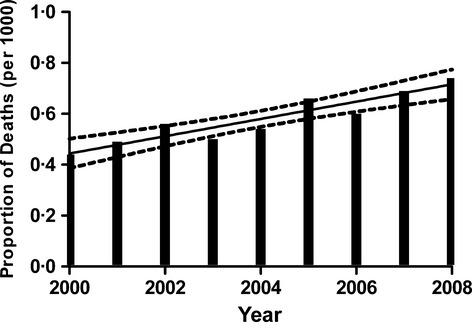
Proportion of death certificates from England on which amyloidosis was included as a cause of death.

According to death certificate data, 0·58 per thousand deaths were attributable to amyloidosis in England between 2000 and 2008. Over the 9-year period, there was a significant increase in the proportion of death certificates on which amyloidosis was included as a cause of death (Fig 2, *R*^2^ 0·86, *P* ≤ 0·001). Given that 21% of patients who were known to the NAC with systemic amyloidosis did not have ‘amyloidosis’ mentioned on their death certificates, we estimated that at least the same proportion of cases may not be reported on death certificates in England generally. Assuming 21% deaths from systemic amyloidosis were not reported on English death certificates between 2000 and 2008, the total number of individuals who died from systemic amyloidosis in England during this time period was 3077 [2543 (79% reported) + 534 (21% unreported)], representing 0·7 per 1000 deaths in the country. Taking only the data for 2008 and using the same calculation (i.e., assuming 21% deaths from systemic amyloidosis were not reported as such), the total number of deaths from systemic amyloidosis in England in 2008 was 429, representing 0·9 per 1000 (429/475 763) deaths.

### Estimate of incidence and prevalence of amyloidosis in England

Referrals to the NAC of patients with systemic amyloidosis (Table IV) doubled over the 9 year study period (*R*^2^ 0·8167, *P* = 0·0008) from 0·2 per 100 000 population in 2001 to 0·4 per 100 000 population in 2008. The incidence increased with age and peaked between 60 and 79 years. A proportion of patients with systemic amyloidosis are not referred to the NAC, and although this proportion appeared to fall substantially between 2000 and 2008 (Table II), it is only possible to reliably estimate the minimum disease incidence from NAC data. The twofold increase in referrals to the NAC between 2000 and 2008 is likely to reflect a combination of increased awareness of the disease and demand for NAC services, as well as improvement in diagnosis. In 2008, 48·2% of the individuals in England in whom amyloidosis was recorded on their death certificate had been assessed at the NAC. Extrapolation to the population of England generally suggests an annual incidence in 2008 of 0·8/100 000 persons. Table V shows the number of patients in England diagnosed with different types of systemic amyloidosis at the NAC. Systemic AL amyloidosis is the most prevalent, with a minimum overall incidence of 0·3 cases per 100 000 population in 2008, which increases to 0·5 per 100 000 when the estimated 51·8% of patients who were not seen at the NAC are included.

**Table IV:** Estimated incidence of systemic amyloidosis in England by age based purely on confirmed diagnoses among patients attending the National Amyloidosis Centre (NAC)

Year	0–19 years	20–29 years	30–39 years	40–49 years	50–59 years	60–69 years	70–79 years	80+ years	Total newly diagnosed cases (Overall estimatedIncidence)	Total alive at NAC/Prevalence*	Total population
2000											
Newly diagnosed cases at NAC (incidence†)	0 (0)	1 (0·01)	2(0·02)	22 (0·3)	31 (0·5)	48 (1·0)	20 (0·5)	5 (0·2)	129 (0·26)	435/0·88	
Population	12 357 900	6 350 000	7 736 000	6 511 400	6 083 300	4 570 300	3 626 900	1 997 300			49 233 100
2001											
NAC	0 (0)	2 (0·03)	15 (0·2)	13 (0·2)	33 (0·5)	44 (0·9)	36 (1·0)	1 (0·05)	144 (0·29)	498/1·00	
Population	12 327 700	6 307 100	7 769 600	6 616 900	6 197 600	4 555 600	3 597 600	2 077 900			49 450 000
2002											
NAC	0 (0)	0 (0)	9 (0·1)	10 (0·1)	44 (0·7)	62 (1·0)	44 (1·0)	6 (0·28)	175 (0·35)	581/1·17	
Population	12 334 200	6 245 000	7 759 700	6 743 000	6 280 600	4 574 000	3 577 600	2 135 000			49 649 100
2003											
NAC	0 (0)	1 (0·01)	6 (0·07)	8 (0·1)	46 (0·7)	62 (1·0)	51 (1·0)	9 (0·41)	183 (0·36)	646/1·29	
Population	12 358 000	6 232 900	7 694 600	6 890 900	6 301 500	4 657 800	3 565 300	2 172 300			49 873 300
2004											
NAC	0 (0)	6 (0·09)	4 (0·05)	20 (0·2)	41 (0·6)	61 (1·0)	41 (1·0)	12 (0·54)	185 (0·37)	710/1·41	
Population	12 365 100	6 318 600	7 575 000	7 040 800	6 309 100	4 742 600	3 555 600	2 203 000			50 109 800
2005											
NAC	1 (0·008)	1 (0·01)	6 (0·08)	16 (0·2)	40 (0·6)	75 (1·0)	47 (1·0)	9 (0·40)	195 (0·38)	788/1·56	
Population	12 350 400	6 488 200	7 459 400	7 217 900	6 313 100	4 836 800	3 563 900	2 236 300			50 466 000
2006											
NAC	0 (0)	3 (0·04)	11 (0·1)	15 (0·2)	34 (0·5)	62 (1·0)	62 (1·0)	13 (0·57)	200 (0·39)	853/1·68	
Population	12 337 600	6 641 400	7 313 000	7 369 200	6 319 700	4 927 200	3 579 100	2 277 000			50 764 200
2007											
NAC	0 (0)	0 (0)	4 (0·05)	18 (0·2)	44 (0·7)	86 (1·0)	89 (2·0)	21 (0·90)	262 (0·51)	966/1·89	
Population	12 351 800	6 826 500	7 142 600	7 501 100	6 217 700	5 138 800	3 607 600	2 320 100			51 106 200
2008											
NAC	3 (0·02)	3 (0·04)	10 (0·1)	17 (0·2)	50 (0·8)	83 (1·0)	82 (2·0)	19 (0·8)	267 (0·51)	1051/2·04	
Population	12 360 600	6 988 300	7 014 300	7 588 900	6 183 100	5 323 800	3 650 400	2 355 200			51 464 600

* Total number of people alive with the disease who have been seen at the NAC per hundred thousand population.

† Newly diagnosed cases per hundred thousand population.

**Table V:** Estimated age-adjusted annual incidence in 2008 of each amyloid type per hundred thousand population in England assuming that all patients with amyloidosis are seen at the National Amyloidosis Centre

	Number of patients (annual incidence per hundred thousand patients)			
Age range (years)	AL amyloidosis	AA amyloidosis	Senile systemic amyloidosis	Hereditary amyloidosis and other
0–19	0	3 (0·03)	0	0
20–29	0	3 (0·04)	0	0
30–39	1 (0·01)	6 (0·08)	0	3 (0·04)
40–49	11 (0·1)	5 (0·06)	0	1 (0·01)
50–59	38 (0·6)	7 (0·1)	0	5 (0·08)
60–69	59 (1·0)	14 (0·2)	1 (0·02)	8 (0·1)
70–79	54 (1·0)	9 (0·2)	10 (0·3)	9 (0·2)
80+	11 (0·5)	1 (0·05)	7 (0·3)	0
Total	174 (0·3)	48 (0·08)	18 (0·03)	26 (0·04)

According to NAC data, there were 435 individuals living in England with systemic amyloidosis in 2000 and 1051 individuals living with the disease in 2008. This apparent increase in prevalence is likely, in part, to reflect improved survival [median (95% confidence interval) 27·6 (16·4–38·9) months in 2000 vs. 45 (43·2–46·7) months in 2008; log rank test for trend *P* = 0·02; Table VI], as well as changing referral patterns.

**Table VI:** Kaplan–Meier survival from the date of diagnosis among patients diagnosed with amyloidosis at the National Amyloidosis Centre by individual year of diagnosis

	Median survival (months)	
Year of diagnosis	Estimate	95% Confidence interval
2000	27·7	16·4–38·9
2001	33·2	16·7–49·7
2002	17·4	11·3–23·5
2003	32·3	20·5–44·1
2004	27·5	16·2–38·8
2005	29·2	18·5–39·8
2006	36·0	20·5–51·6
2007	33·9	23·1–44·7
2008	45·0	43·2–46·7
Overall	31·8	27·4–36·2

Log rank (Mantel Cox) for trend *P* = 0·023.

### Regional differences in death rates and referrals to the NAC

**Fig. 3:**
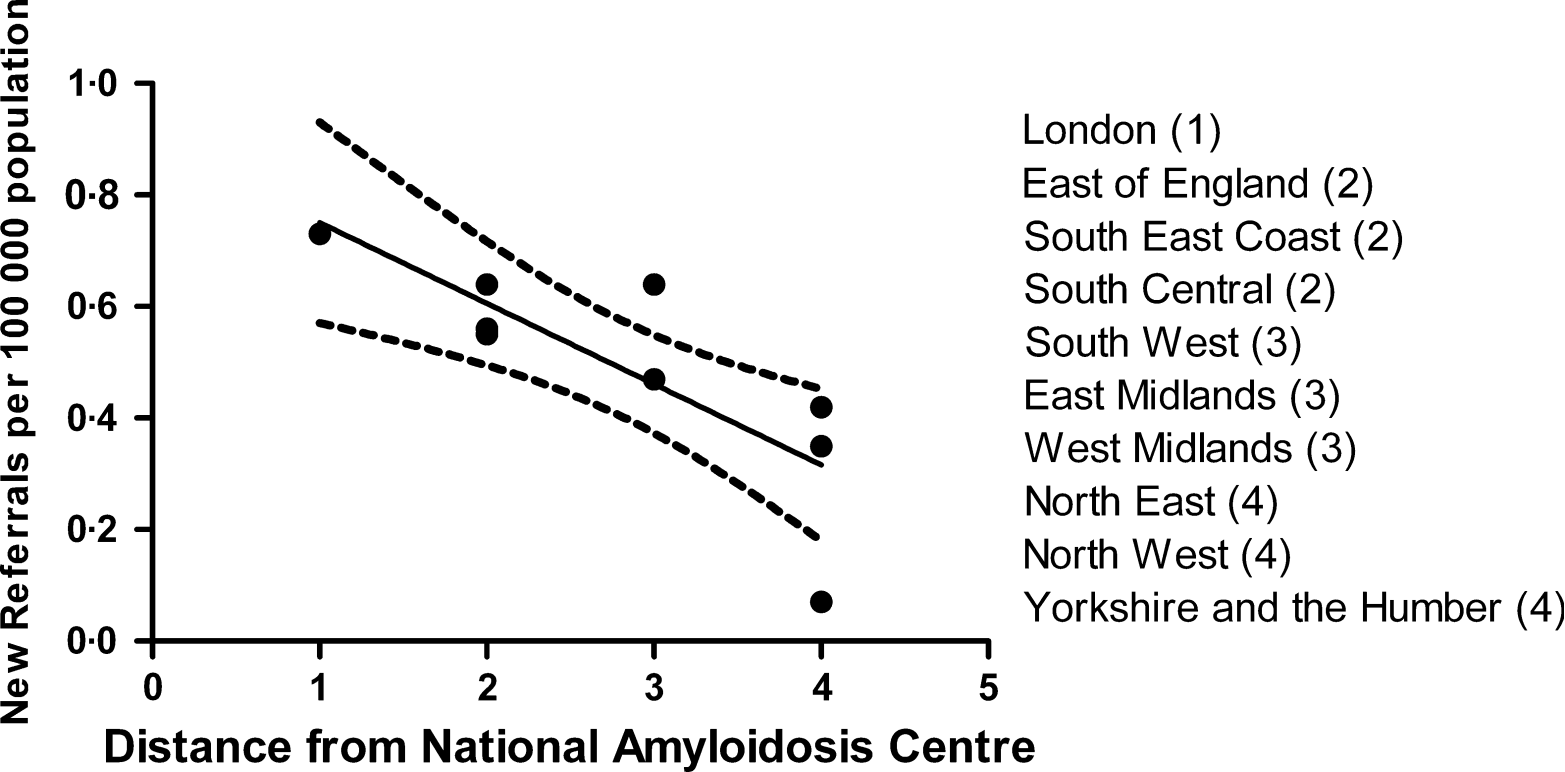
Apparent incidence of amyloidosis in 2008 stratified by Strategic Health Authority, derived solely from new referrals to the National Amyloidosis Centre (NAC). The incidence appears to fall as distance from the NAC increases (*R*^2^ = 0·64, *P* = 0·005).

The regional differences across England in reported deaths from amyloidosis in 2008 are shown in Table VII. The proportion of deaths ranged from a minimum of 0·55/1000 in Yorkshire and the Humber to a maximum of 0·97/1000 in the South West. There were significantly less deaths reported in Yorkshire and the Humber compared to the East of England, South Central and the South West (*P* < 0·01). There was no correlation between the proportion of deaths from amyloidosis and distance from the NAC. The number of new referrals to the NAC did vary between strategic health authorities (SHAs) however, with a greater number of patients being referred from SHAs located closer to the NAC (Fig 3). It would appear that nearly all patients who died from systemic amyloidosis in London and the East of England regions had been seen at the NAC. These regions are therefore likely to offer the greatest accuracy for estimating disease incidence. The minimum estimated disease incidence in these two regions respectively, based on new patient referrals to the NAC, was 0·73 and 0·56/100 000 population, corroborating earlier calculations of disease incidence.

**Table VII:** Total deaths and National Amyloidosis Centre deaths from amyloidosis and incidence based on new referrals to the NAC in 2008 by Strategic Health Authority

Strategic Health Authority	ONS total deaths	ONS population estimates	Amyloidosis on death certificate	Proportion of deaths due to amyloidosis (per thousand)	Deaths from amyloidosis (per 100 000 population)	New patients diagnosed with amyloid at NAC	Incidence of amyloidosis (based on newly diagnosed NAC cases) per 100 000	Number of deaths in NAC patients
North East	27 386	2 570 600	20	0·73	0·77	2	0·07	4
North West	70 740	6 874 100	45	0·63	0·65	24	0·35	24
Yorkshire and the Humber	50 539	5 217 500	28	0·55	0·54	22	0·42	13
East Midlands	42 296	4 429 400	25	0·59	0·56	21	0·47	8
West Midlands	52 318	5 408 400	33	0·63	0·61	35	0·64	13
East of England	52 689	5 717 400	49	0·93	0·85	32	0·56	50
London	50 476	7 668 300	42	0·83	0·54	56	0·73	38
South East Coast	42 537	4 309 400	26	0·61	0·6	24	0·55	16
South Central	33 380	4 059 100	33	0·98	0·81	26	0·64	24
South West	53 402	5 210 400	52	0·97	0·99	25	0·47	27
Total	475 763	51 464 600	353	0·74	0·68	267	0·52	217

ONS, Office of National Statistics; NAC, National Amyloidosis Centre.

## Discussion

Information regarding the epidemiology of systemic amyloidosis is scarce. The most robust study (Kyle *et al*, 1992) reported the incidence to be 8·9 per million person years. This study used patient record data from Olmstead County, USA to identify patients diagnosed with the disease between 1950 and 1989, and incidence was extrapolated on the basis of population data for the whole of the USA. The authors noted an increase in incidence across the time period (Kyle *et al*, 1992). The findings from the Olmstead County study have not been validated or updated; furthermore, the epidemiology of systemic amyloidosis has not previously been studied in England. A report from Boston University used death certificate data to estimate the number of deaths from systemic AL amyloidosis. Although the diagnostic criteria for AL amyloidosis were somewhat unreliable in this study, the estimated incidence based on mortality data was 4·5/100 000 (Simms *et al*, 1994). Imaizumi (1989) used death certificates to estimate the rate of death from systemic amyloidosis in Japan, expressed as the number of cases with amyloid on death certificates per 100 000 population alive during that year. They reported an increase in death rate from amyloid among males from 0·022/100 000 in 1969 to 0·178/100 000 in 1992 (Imaizumi, 1989).

Death certificates have previously been used to estimate incidence in a number of rare diseases (Marin *et al*, 2011). Death certificate data is deemed a reliable way of estimating disease incidence only in conditions which are well defined, rarely misdiagnosed, and consistently fatal without changing survival (Marin *et al*, 2011). The sensitivity of mortality data for predicting disease incidence in systemic AL amyloidosis has not been systematically determined; on the one hand, there are well defined diagnostic criteria, it has a high mortality and short survival (Simms *et al*, 1994), on the other hand, it may be misdiagnosed (Lachmann *et al*, 2002) and survival has steadily been improving probably as a result of increased use of novel drug therapies (Kumar *et al*, 2010; Kastritis *et al*, 2012; Venner *et al*, 2012), introducing uncertainty into calculations of disease incidence. In this study, we determined that amyloidosis was present on the death certificates of 79% of individuals with known systemic amyloidosis in England and 82·5% of individuals with known systemic AL amyloidosis. Using the information from death certificates, an estimated 0·58/1000 deaths in England were attributable to amyloidosis, with a significant increase reported over the decade. Among those reviewed at the NAC who died, 80% had AL amyloidosis. One would estimate therefore that *c*. 0·46/1000 deaths in England are attributable to AL amyloidosis. Patient survival increased substantially between 2000 and 2008; one would therefore be reluctant to estimate disease incidence from death certificate data alone.

Individual level data for patients reviewed at the NAC are likely to be extremely reliable, particularly with respect to presence and type of amyloid. Only 48·2% of patients with systemic amyloidosis who died in 2008 had been seen at the NAC however, clearly suggesting that not all patients with systemic amyloidosis in England are seen at the centre. An estimate of incidence of systemic amyloidosis that is calculated solely on the basis of NAC cases would undoubtedly be an underestimate; however, 0·4 per 100 000 population in 2008 can reliably be considered a minimum disease incidence. The true incidence of systemic amyloidosis is likely to be approximately double this Figure, *c*. 0·8 per 100 000, although once again, this may be an underestimate given that 21% of patients with systemic amyloidosis do not have amyloidosis on their death certificates. Without a national register of all cases of amyloid however, these limitations cannot be addressed.

The incidence, and/or the recognition of systemic amyloidosis appear to be increasing. Data from Olmstead County showed an apparent rise in incidence of AL amyloidosis during the last decade of their analyses. In the present study, there was a significant rise each year in the number of individuals with amyloidosis on their death certificate, accompanied by a parallel rise in the number of patients with systemic amyloidosis assessed at the NAC throughout the study period. This apparent rise may reflect an actual increase in disease incidence but could equally well be attributed to better awareness of the disease itself and/or existence of a national referral centre as well as improved diagnostic techniques.

## Study limitations

From a combination of NAC data and death certificate data we have been able to robustly calculate the sensitivity of death certificates in reporting systemic amyloidosis. Unfortunately, we were unable to calculate the specificity of death certificate data for reporting systemic amyloidosis because we do not have further clinical information on many of the relevant individuals although, given that amyloid histology is required for diagnosis and is extremely specific, one can assume that specificity is likely to be very high. We have used the number of deaths among amyloidosis patients who were not seen at our centre to make an estimate of the total number of patients with amyloidosis in England; however, we do not know the proportion of patients who are diagnosed with amyloidosis but never seen at the NAC that have amyloidosis on their death certificates. It is conceivable that patients with systemic amyloidosis who do attend the NAC are more likely to have amyloidosis on their death certificate than those with systemic amyloidosis who do not attend the NAC.

## Conclusion

Death certificates are a reasonably sensitive method for detecting the proportion of deaths attributable to systemic amyloidosis in England. The proportion of individuals diagnosed with systemic amyloidosis who are seen at the UK NAC is increasing each year. We estimate that the annual incidence of systemic amyloidosis in 2008 was approximately 0·8 per 100 000 population.
